# Comparison of two multiplexed technologies for profiling >1,000 serum proteins that may associate with tumor burden

**DOI:** 10.12688/f1000research.53364.1

**Published:** 2021-06-28

**Authors:** Annie Ren, Ioannis Prassas, Vijithan Sugumar, Antoninus Soosaipillai, Marcus Bernardini, Eleftherios P Diamandis, Vathany Kulasingam

**Affiliations:** 1 Department of Laboratory Medicine and Pathobiology, University of Toronto, Toronto, Ontario, Canada; 2 Lunenfeld-Tanenbaum Research Institute, Mount Sinai Hospital, Toronto, Ontario, Canada; 3 Department of Pathology and Laboratory Medicine, Mount Sinai Hospital, Toronto, Ontario, Canada; 4 Division of Gynecologic Oncology, University Health Network, Toronto, Ontario, Canada; 5 Department of Clinical Biochemistry, University Health Network, Toronto, Ontario, Canada

**Keywords:** Proteomics, cancer biomarkers, proximity extension assay, antibody array, multiplex, immunoassay, ovarian cancer

## Abstract

**Background:** In this pilot study, we perform a preliminary comparison of two targeted multiplex

proteomics technologies for discerning serum protein concentration changes that may correlate to tumor burden in ovarian cancer (OC) patients.

**Methods**: Using the proximity extension assay (PEA) and Quantibody® Kiloplex Array (QKA), we measured >1,000 proteins in the pre-surgical and post-surgical serum from nine OC patients (N=18 samples). We expect that proteins that have decreased significantly in the post-surgical serum concentration may correlate to tumor burden in each patient. Duplicate sera from two healthy individuals were used as controls (N=4 samples). We employed in-house ELISAs to measure five proteins with large serum concentration changes in pre- and post-surgical sera, from four of the original nine patients and the two original controls.

**Results:** Both platforms showed a weak correlation with clinical cancer antigen 125 (CA125) data. The two multiplexed platforms showed a significant correlation with each other for >400 overlapping proteins. PEA uncovered 15 proteins, while QKA revealed 11 proteins, with more than a two-fold post-surgical decrease in at least six of the nine patients. Validation using single enzyme-linked immunosorbent assays (ELISAs) showed at least a two-fold post-surgical decrease in serum concentration of the same patients, as indicated by the two multiplex assays.

**Conclusion:** Both methods identified proteins that had significantly decreased in post-surgical serum concentration, as well as recognizing proteins that had been implicated in OC patients. Our findings from a limited sample size suggest that novel targeted proteomics platforms are promising tools for identifying candidate serological tumor-related proteins.  However further studies are essential for the improvement of accuracy and avoidance of false results.

## List of abbreviations

ACVR1, activin receptor type 1

BCAM, basal cell adhesion molecule

BMP9, bone morphogenetic protein 9

BSA, bovine serum albumin

CA125, cancer antigen 125

COMP, cartilage oligomeric matrix protein

CV, coefficients of variation

CYFRA21-1, keratin type I cytoskeletal 19

PARP-1, poly [ADP-ribose] polymerase 1

ELISA, enzyme-linked immunosorbent assay

EPCAM, epithelial cell adhesion molecule

FC, fold change

FOLR1, folate receptor 1

GYN, gynecologic

HE4, human epididymis protein

IFNL1, interferon lambda 1

IgG, immunoglobulin G

KLK, kallikrein

KRT19, keratin type I cytoskeletal 19

LLD, lower limit of detection

LOA, limits of agreement

MS, mass spectrometry

MSLN, mesothelin

NAMPT, nicotinamide phosphoribosyltransferase

NPX, normalized protein expression

OC, ovarian cancer

PAEP, glycodelin

PCR, polymerase chain reaction

PEA, proximity extension assay

QKA, Quantibody ^®^ KiloplexArray

SLAM, signaling lymphocytic activation molecule

STIP1, stress-induced phosphoprotein 1

TLR, toll-like receptor

TROP2, trophoblast antigen 2

UHN, University Health Network

## Introduction

Advancements in omics technologies have been instrumental in illuminating the heterogenous traits of cancer cells that harbor distinct genetic, epigenetic and/or phenotypic fingerprints. ^1^ The further understanding of this intra- and inter-tumoral heterogeneity has propelled a new era of precision medicine, which seeks to personalize clinical practices, such as disease prognosis, treatment selection and monitoring treatment efficacy, according to the unique molecular makeup of each tumor. ^2, 3^ Among the multitudes of omics technologies, examining the proteome is the ultimate lens into the functional units of tumor cells. The blood proteome is a particularly rich mine for unearthing information on the pathophysiological traits of an individual. Tumors secrete a medley of tumor-derived proteins into the blood, which could be quantified to serve as proxies of tumor status. The concentration of tumor-related proteins is further propagated with highly increased expression during cancer progression. The detection and measurement of proteins in blood plasma/serum is thus very advantageous for mapping tumor activity in each patient. This would enable the discovery and validation of new biomarkers for the prediction of disease progression, treatment response, and effective personalized treatment for patients.

The proteomics technique, mass spectrometry (MS), conventionally centers around a system-wide, unbiased approach to protein detection. However, pre-analytical and analytical variation, detection of low abundance proteins, and the depth of proteome coverage remain major hurdles for the MS-based methods, in relation to analyzing complex biological fluids. ^4^ Complementing a mounting interest in precision medicine is a timely advent of new, targeted proteomics platforms, aimed at aiding biomarker discovery with enhanced sensitivity, specificity, multiplexing, and throughput. These immunoassay-based multiplex technologies could potentially screen thousands of proteins, while detecting miniscule amounts of clinically relevant, tumor-associated proteins in the plasma or serum of individual patients. ^5^ We previously described such tumor-associated proteins identified in cancer patients as personalized tumor markers, where each marker is highly specific and sensitive for cancer detection in 5-30% of patients. ^6, 7^ Cancer screening individual patients against a repertoire of personalized tumor markers, by using a targeted proteomics method could identify a distinct protein signature that best characterizes the mosaicism of each unique tumor. This would help guide treatment and surveillance.

In this study, we sought to conduct preliminary analyses examining the technical feasibility of two targeted multiplex proteomics technologies for detecting changes in serum protein concentrations that could correspond to tumor burden in individual patients, using advanced ovarian cancer (OC) as a model. OC is often diagnosed at late stages, where the survival rate is only 30%. ^8^ Clinical practices for diagnosing, monitoring, and guiding treatment are reliant on imaging modalities and the serum-based biomarker, CA125. ^8^ However, genomic studies have unveiled a diverse tumor mutational landscape, with advanced OC deriving from numerous genetic mutations and intra-individual genetic heterogeneity heavily accounting for relapse and acquired chemoresistance. ^9^ The indisputable evidence on OC genetic heterogeneity espouses the need to build a library of personalized tumor markers that may better represent the diverse tumors found in patients.

Herein, we performed an initial assessment of the technical reliability of two commercially available multiplex proteomics technologies, the proximity extension assay (PEA) and the Quantibody ^®^ KiloplexArray (QKA), for delineating changes in serum protein concentrations that may correlate to OC tumor burden. The platforms used in this study reflect two different ways of improving the sensitivity and throughput of traditional sandwich-type ELISAs. The PEA technology is based on immuno-polymerase chain reaction (PCR) and uses dual-recognition antibodies that are labelled with complementary oligonucleotides to generate quantitative PCR signals corresponding to protein concentration. The QKA works by precisely printing captured antibodies on glass slides and leveraging nanofluidics to perform 1000 micro-ELISAs in a single experiment. Over 1000 proteins analyzed by each platform span a wide biological and pathological relevance, such as their involvement in pro-inflammatory responses, immune response, cell regulation, and tumorigenesis. To evaluate these technologies, we employed each method to measure ≥1000 proteins in paired serum samples collected pre- and post-surgically from OC patients. In addition to performing technical comparisons for data derived from the ≥1000 proteins and >400 overlapping targets, we selected proteins that had the most decrease in serum after surgery, according to each proteomics method. We speculate significant decrease in serum proteins following the tumor debulking surgery, may be associated with changes in tumor burden in the patient. We further conducted orthogonal validation for five of the selected proteins using verified, in-house, single target ELISAs, to evaluate the reliability and accuracy of the platforms. Our study takes a comparative approach to serum protein profiling in cancer by using two different targeted proteomics methods. These methods offer a preliminary glimpse into the strengths and limitations of each technology for the identification of serum proteins that may correlate to tumor burden, and individualized serum protein signatures that could provide information on tumor heterogeneity.

## Methods

### Sample collection

Serum samples from nine advanced high-grade serous carcinoma OC patients were collected one week before optimal debulking surgery, and at four weeks after surgery (n = 18). These samples were retrospectively obtained from the University Health Network (UHN) Gynecologic (GYN) Blood Biobank. The patient inclusion criteria included patients who showed pre-surgical clinical CA125 values of at least 1000 U/mL, in order to select for patients with high tumor load prior to surgery, as well as those who received primary debulking surgery followed by six cycles of platinum/taxane-based chemotherapy as their first-line treatment. Samples were collected and stored at -80 °C, prior to analysis with a standardized protocol to avoid systemic biases. One serum sample from a healthy male and female were split into two identical aliquots (n = 4) for use as controls.

### Proximity extension assay

Sera from OC patients and controls were analyzed for intra-individual relative protein concentration changes using the multiplex PEA technology (Olink Proteomics, Uppsala, Sweden). A total of 1073 unique proteins were assayed across 13 panels (92-plex) , which span a broad dynamic range of eight to 10-log with the sensitivity of sub fg/mL to pg/mL. We have previously described the workflow of the PEA technology. ^10^ In brief, 1μL of serum sample was incubated with 92 pairs of oligonucleotide-labeled antibodies. Upon binding of both antibodies to the target, the single-stranded DNA ends hybridize and act as a template for DNA polymerase-dependent extension. To ensure the analytical quality of the samples that ran in singlicates, three types of internal controls (for sample quality, immunoglobulin G (IgG) binding, oligonucleotide annealing, and PCR amplification) are spiked into each sample prior to conducting the assay. Control sera in duplicate, negative control samples in triplicate, and inter-plate sera in triplicate are also included in each assay plate to control for signal specificity, inter-well variation and inter-plate variation. The DNA amplicon which results from the extension step, is detected with qPCR on the BioMark HD Real-Time PCR System (Fluidigm, CA, USA). The signal from each unique amplicon is proportional to protein concentration [expressed as a relatively quantitative, arbitrary unit on a log2 scale termed, normalized protein expression (NPX)]. NPX values are determined by normalizing each signal to the median signal per plate. To minimize bias, the technical personnel were blinded to the sample status and only intra-plate NPX value comparisons were made.

The workflow of some panels uses serum at a pre-specified dilution (Cardiometabolic: 1:2025; Cardiovascular III: 1:100; Development: 1:100; Metabolism: 1:10) to detect high abundance proteins in expected physiological and pathological ranges. A very low post-surgical fold change (FC) in CA125 (in Oncology panel) was observed, when we ran undiluted sera as recommended by the manufacturer ( **Underlying data**, ^11^ therefore only the pre- and post-surgical sera with 1:10 dilution for the Oncology panel were performed. We identified three proteins from this panel [CA125, human epididymis protein (HE4), and folate receptor 1 (FOLR1)] that exhibited signs of the Hook effect in the undiluted analysis, as the pre-surgical serum concentrations were higher in the 1:10 dilution versus undiluted analysis. Therefore, we used the pre- and post-surgical serum levels of CA125, HE4, and FORL1 that were obtained from the 1:10 dilution, for the continuation of our analysis.

### Quantibody ^®^ human kiloplex array

We employed the QKA (RayBiotech, GA, USA) to concurrently measure 1000 proteins in sera from OC patients and controls. The biological relevance of the 1000 target molecules is mainly inflammation-related. The platform, which combines 25 nonoverlapping 40-plex arrays to perform 1000 sandwich ELISAs simultaneously, possesses a dynamic range of around 3- to 4-log concentration and sensitivity ranging from 1 pg/mL to 100 ng/mL. The workflow of the array was previously described. ^12^ In brief, each array included protein standards to generate a standard curve for absolute protein quantitation. The samples were analyzed in technical quadruplicates, and the technical personnel were blinded to the sample status. We diluted 750μL of serum four-fold to a final volume of 3 mL for use in all 25 arrays. Capture antibodies were bound to the glass surface in the multiplex. After blocking and sample incubation, nonspecific binding was washed away, and a biotin-labeled detection antibody was subsequently added. Finally, Streptavidin-conjugated Cy3 equivalent dye was added to yield fluorescent signals, which were read using a microarray laser scanner (GenePix 4200A, Molecular Dynamics, Sunnyvale, CA, USA). The Q-Analyzer software (Raybiotech) was used to compute protein concentration, after accounting for intra- and inter-slide normalization.

### Selection of proteins with a post-surgical decrease in serum concentration

We analyzed the data from each multiplex proteomics method by first calculating the FC in serum concentration from the pre-surgical to post-surgical sample for each protein and patient. For all proteins, concentrations that were below the lower limit of detection (LLD) of the method were normalized to equal the value of the LLD. As a reference to the technical variation, we also calculated the FC between the technical duplicate measurements of the control samples for each technology. In the control samples, proteins with more than two-FC between the duplicate measurements were removed. In at least six of the nine patients, proteins with pre-specified two-fold or higher decrease in their post-surgical serum concentrations were selected as biomarker candidates.

### ELISA procedures

In-house single target ELISA assays previously developed were used to validate the post-surgical serum concentration changes in the five proteins [kallikrein-5 (KLK5), KLK6, KLK7, KLK10, and KLK11] that were observed in the same four of the nine patients (Patient 3, 6, 7, 8 for the PEA analysis and patient 1, 3, 6, 7 for the QKA), and the two controls. The type and sources of all antibodies used in the immunoassays and the assay workflow are previously described. ^13^ In brief, sandwich format immunoassays for KLK5, 6, 7, and 10 employed monoclonal-monoclonal antibody configurations. We coated each well in microtiter plates with 500ng of monoclonal antibody in 100μL coating buffer (50 mmol/L Tris-HCl, pH 7.8). We incubated the plates overnight before washing (10 mmol/L Tris-HCl, pH 7.4, containing 150 mmol/L NaCl and 0.5 mL/L Tween 20). Next, we added 50 μL recombinant protein standards or samples diluted in 6% bovine serum albumin (BSA), along with 50 μL assay buffer (6% BSA containing 25 mL/L normal mouse serum, 100 mL/L normal goat serum, and 10 g/L bovine IgG). After incubation and wash, we added 50ng of our in-house biotinylated monoclonal detection antibody diluted in 100 μL assay buffer, as previously described. ^13^ Following incubation and wash, we added 5 ng alkaline phosphatase–conjugated streptavidin diluted in 100 μL 6% BSA. Final round of incubation and wash was followed by, incubation of each well with 100 μL diflunisal phosphate solution (0.1 mol/L Tris-HCl, pH9.1, containing 1 mmol/L diflunisal phosphate, 0.1 mol/LNaCl, and 1 mmol/L MgCl _2_). Finally, we added 100 μL developing solution (1 mmol/L Tris, 0.4 mol/L NaOH, 2 mmol/L TbCl3, and 3 mmol/L EDTA), before measuring the fluorescence with a time-resolved fluorometer, The EnVision ^®^ 2105 multimode plate reader (PerkinElmer Life Science, MA, USA). Standard curve and protein concentration calculations were performed automatically, as previously described. ^14^ We used a monoclonal-polyclonal ELISA configuration for measuring KLK11. The assay followed the same procedures, except we added 50 ng of our in-house biotinylated polyclonal antibody. ^13^

### Statistical analysis

Statistical analyses were performed using the GraphPad Prism software (version 4.02). Pearson correlation coefficients were computed to evaluate the correlation between serum protein concentrations obtained from QKA, PEA and ELISA. P values of less than 0.05 were considered statistically significant. Bland-Altman plots were used to assess the agreement of measurements between two methods. For the latter, we calculated the mean difference, or bias, between measurements by the two methods, and the 95% limits of agreement (LOA) using the formula, bias ± 1.96* Standard Deviation (SD). Agreement is determined by how close the bias is to zero.

## Ethical considerations

Serum samples from OC patients with high-grade serous carcinoma were retrospectively obtained from the UHNGYN Blood Biobank with approval by the Research Ethics Board (REB) of UHN, Toronto, Canada (REB #10-0591). All eligible patients attending the GYN Oncology Clinics at the Princess Margaret Cancer Centre (Toronto, ON, Canada) are approached at their initial appointment and offered the opportunity to participate in the UHN GYN Blood Biobank. The purpose of the biobank, procedures, and schedule of collections are reviewed and explained thoroughly, aiding in written informed consent (as per UHN Research Ethics Board guidelines and UHN REB-approved consent document). Upon consent, additional blood samples are collected for future research at the same time as clinical bloodwork. These samples are linked to high quality de-identified clinical data.

## Results

### Concordance of PEA and QKA data with clinical assessment of CA125

**Figure 1.**
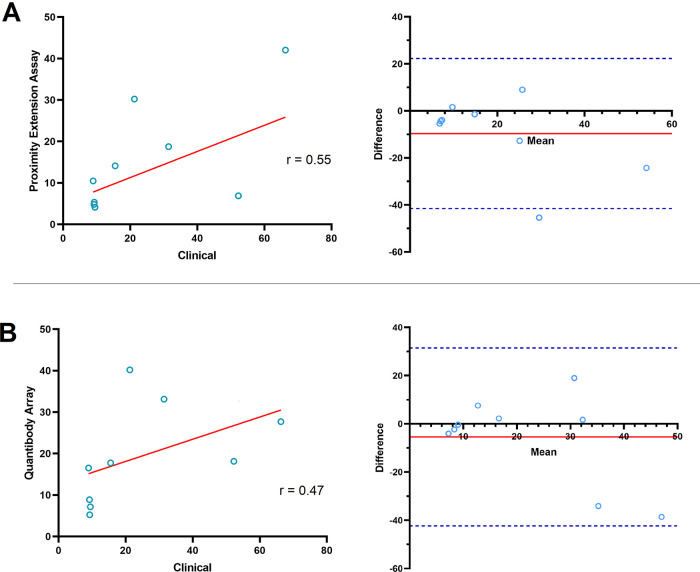
Comparison between CA125 values obtained by clinical assessment and multiplex proteomics technologies in 9 ovarian cancer patients. A) Proximity extension assay (PEA): (Left) Scatterplot analysis of the post-surgical CA125 fold change assessed by PEA technology and clinical assay showed a weak correlation (Pearson correlation coefficient (r) = 0.546, P = 0.066). Solid red line in scatterplots shows the line of best fit. (Right) Bland-Altman plot of the post-surgical fold change in CA125 measured by PEA and the clinical assay. Solid red line shows the mean difference or bias in fold change measurements (−9.64). Dashed blue lines show the upper and lower limit of agreement (LOA; Mean ± 1.96 SD; ranges from -41.55 to 22.27 difference in fold change). B) Quantibody array: (Left) Similarly, there is a weak correlation between the post-surgical CA125 fold change measured by Quantibody array and clinical assay (r = 0.470, P = 0.202). (Right) Bland-Altman plot of the post-surgical CA125 fold change assessed by Quantibody array and clinical assay. Solid red line exhibits bias (−5.43). Dashed blue lines show the upper and lower LOA (−42.31 to 31.46).

We compared the post-surgical FC in CA125 reported by the clinic and the two proteomics platforms in the nine OC patients ( Figure 1). Scatterplot analysis of FC assessed by PEA and the clinical assay showed a weak correlation [ Figure 1A, Pearson correlation coefficient (r) = 0.546, P = 0.066]. Bland-Altman plot for the two methods showed a bias of −9.64 in FC. Signifying PEA showed a lower post-surgical FC in CA125 overall. Measurement in one patient (P3) resided outside of the LOA (−41.55 to 22.27 difference in FC). Similarly, there is a weak correlation between post-surgical CA125 FC measured by QKA and clinical assay ( Figure 1B, r = 0.470, P = 0.202). Bland-Altman analysis of QKA versus clinical data showed a bias of −5.43. The LOA ranged from −42.31 to 31.46, with all measurements within limit.

### Qualitative comparative analysis of PEA and QKA measurements

**Figure 2.**
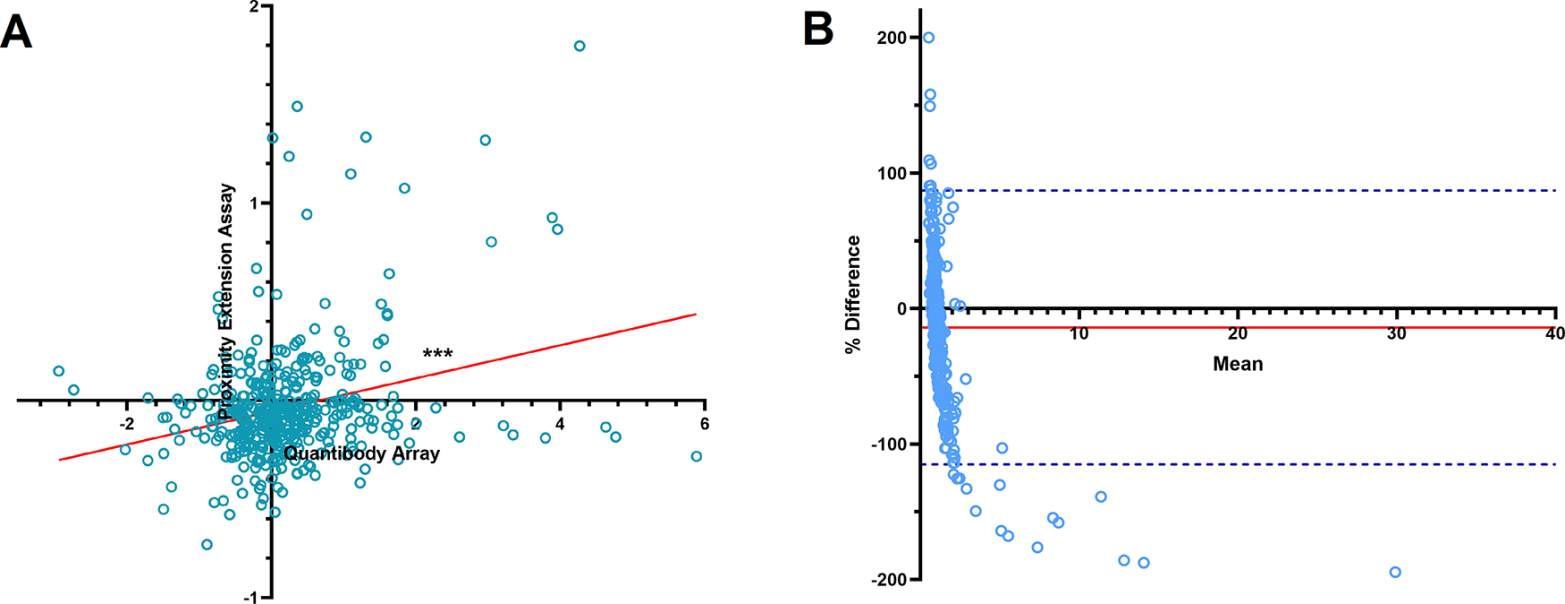
Comparative analysis of 443 proteins measured by proximity extension assay (PEA) and Quantibody array. A) Scatterplot showed a significant correlation between the post-surgical protein fold changes assessed by PEA technology and Quantibody array (Pearson correlation coefficient (r) = 0.275, ***P < 0.0001). Solid red line shows the line of best fit. B) Bland-Altman plot of the post-surgical protein fold changes measured by PEA and Quantibody array. Solid red line shows the mean % difference or bias in fold change measurements (−13.95%). Dashed blue lines show the upper and lower limit of agreement (Mean ± 1.96 SD; −115.0% to 87.11% difference in fold change). The difference in measurements are shown as percentages to better visualize the large range of data.

We mined the target analytes of the PEA and QKA platforms and identified 444 overlapping proteins **(Extended data**). ^15^ Scatterplot analysis of the post-surgical FC in the 444 proteins from nine OC patients, showed a weak but significant correlation between the methods (r = 0.275, P < 0.0001) ( Figure 2A). Bland-Altman plot showed a minimal bias of −0.68 in the FC (-13.95% difference) between the methods ( Figure 2B). The LOA ranged from −7.84 to 6.47 difference in the FC between the methods (−115.0% to 87.11% difference), where 94.3% of protein measurements were within this range. Differences in measurements are shown as percentages due to the large data range.

### Identification of proteins with post-surgical decrease based on separate and overlapping analysis of PEA and QKA datasets

The PEA analysis identified 15 proteins (including CA125) that had a two-fold decrease between the pre-surgical to post-surgical samples, in six out of the total of nine patients (66.7-100%) ( Table 1). Thirteen proteins had at least five-fold decrease in two of the seven patients (22.2-77.6%) ( Table 1). Nine proteins decreased by at least ten-fold in one of the five patients (11.1-55.6%) ( Table 1). CA125, FOLR1, and keratin type I cytoskeletal 19 (KRT19) showed the highest median post-surgical fold decrease (over 10-fold) ( Table 1).

**Table 1.** Proximity extension assay analysis. Heatmap of post-surgical fold decrease in protein serum concentration in 9 ovarian cancer patients.

	Ovarian Cancer									Healthy controls ^*^			
	P1	P2	P3	P4	P5	P6	P7	P8	P9	C1	C2	Median FD ^**^ in patients	Number of patients with >2 FD ^**^
**Cancer antigen 125 (CA125)**	4.1	4.8	6.9	42.0	10.5	5.4	18.8	30.2	14.1	0.9	1.0	10.5	9
**Folate receptor 1 (FOLR1)**	20.4	1.6	4.6	1.2	43.7	10.5	21.1	22.2	2.5	1.0	0.8	10.5	7
**Keratin type I cytoskeletal 19 (KRT19)**	12.8	2.4	10.2	0.9	17.2	12.9	8.4	16.5	3.4	0.6	0.6	10.2	8
**Kallikrein-10 (KLK10)**	8.4	2.5	11.5	1.0	3.4	8.7	19.4	16.0	0.9	1.0	0.6	8.4	7
**Mesothelin (MSLN)**	4.8	1.6	2.2	1.3	5.7	6.6	12.4	7.4	2.7	1.0	0.8	4.8	7
**Poly [ADP-ribose] polymerase 1 (PARP-1)**	14.9	1.3	1.6	2.5	15.9	1.3	4.6	13.0	4.9	1.2	1.3	4.6	6
**Glycodelin (PAEP)**	5.0	4.1	3.8	6.3	8.1	2.3	4.5	11.3	4.0	0.5	0.6	4.5	9
**Kallikrein-11 (KLK11)**	4.1	1.5	4.3	2.9	4.3	3.8	5.9	5.3	1.7	1.1	0.8	4.1	7
**Kallikrein-6 (KLK6)**	3.2	1.3	7.4	1.0	3.0	6.1	4.6	15.0	1.0	0.9	0.8	3.2	6
**Interferon lambda-1 (IFNL1)**	5.9	2.8	1.9	2.1	3.1	3.4	14.5	4.1	1.7	0.8	0.8	3.1	7
**Basal cell adhesion molecule (BCAM)**	6.5	1.3	2.1	1.4	2.9	4.3	4.4	5.5	1.1	0.9	1.0	2.9	6
**Epithelial cell adhesion molecule (EPCAM)**	1.3	2.5	3.0	2.1	2.3	5.3	1.5	8.0	3.2	1.0	0.9	2.5	7
**Kallikrein-8 (KLK8)**	3.2	1.2	3.3	1.4	2.4	3.5	2.0	3.5	1.3	1.0	0.8	2.4	6
**Trophoblast antigen 2 (TROP2)**	8.8	2.0	1.1	1.3	1.4	8.1	6.4	6.8	2.1	0.9	0.8	2.1	6
**Human epididymis protein 4 (HE4)**	3.2	1.4	1.6	2.1	2.6	2.1	3.8	2.0	2.4	1.1	0.8	2.1	6

* Fold change between technical duplicate measurements of healthy controls is shown.

** FD, Fold decrease in protein serum concentration post-surgery.

Grey highlight means protein decreased by at least five-fold in some patients.

Blue highlight means protein decreased by at least ten-fold in some patients.

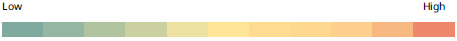

The QKA data yielded 11 proteins that decreased by at least two-fold in six of the nine patients (66.7-100%) ( Table 2). Ten of the proteins ( Table 2 **)** decreased by at least five-fold, in one of the nine patients (11.1-100%). Five proteins ( Table 2) decreased by at least ten-fold, in one of the five patients (11.1-66.7%). CA125, KLK11, FOLR1, and KLK5 showed the highest median post-surgical fold decrease ( Table 2).

**Table 2.** Quantibody Kiloplex Array analysis. Heatmap of post-surgical fold decrease in protein serum concentration in 9 ovarian cancer patients.

	Ovarian Cancer									Healthy controls ^*^			
	P1	P2	P3	P4	P5	P6	P7	P8	P9	C1	C2	Median FD ^**^ in patients	Number of patients with >2 FD ^**^
**Cancer antigen 125 (CA125)**	7.2	8.9	18.2	27.7	16.5	5.2	33.1	40.2	17.8	0.5	0.3	17.8	9
**Kallikrein 11 (KLK11)**	9.3	0.4	11.8	5.4	1.6	30.4	4.9	41.8	5.6	1.9	1.7	5.6	7
**Folate receptor 1 (FOLR1)**	18.3	0.6	4.6	0.1	33.4	6.6	4.9	32.4	1.0	0.0	0.0	4.9	6
**Kallikrein 5 (KLK5)**	14.8	1.8	2.5	0.1	7.0	19.1	3.4	10.4	4.5	1.7	1.9	4.5	7
**Renin**	5.1	2.1	0.8	1.3	0.8	2.8	6.2	5.1	2.8	1.9	1.3	2.8	6
**Activin Receptor Type 1 (ACVR1)**	1.6	2.8	0.8	3.0	4.6	2.5	5.3	1.3	4.3	0.2	0.0	2.8	6
**Nicotinamide phosphoribosyltransferase (NAMPT)**	3.7	1.9	4.8	2.5	0.9	2.7	4.2	5.0	1.5	1.2	0.0	2.7	6
**Signaling lymphocytic activation molecule (SLAM)**	5.3	2.7	5.1	0.9	0.7	2.8	2.1	2.7	1.6	0.0	0.0	2.7	6
**Kallikrein 7 (KLK7)**	9.9	1.4	1.7	2.3	0.6	3.6	3.8	2.6	4.7	1.4	0.6	2.6	6
**Cartilage oligomeric matrix protein (COMP)**	3.9	2.6	2.3	1.3	1.1	2.5	3.4	2.1	1.7	0.9	1.0	2.3	6
**Toll-like receptor 1 (TLR1)**	12.0	2.0	1.4	1.2	0.7	2.3	2.2	4.3	4.6	1.8	0.9	2.2	6

* Fold change between technical duplicate measurements of healthy controls is shown.

** FD, Fold decrease in protein serum concentration post-surgery.

Grey highlight means protein decreased by at least five-fold in some patients.

Blue highlight means protein decreased by at least ten-fold in some patients.

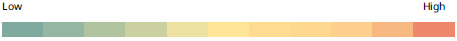

**Figure 3.**
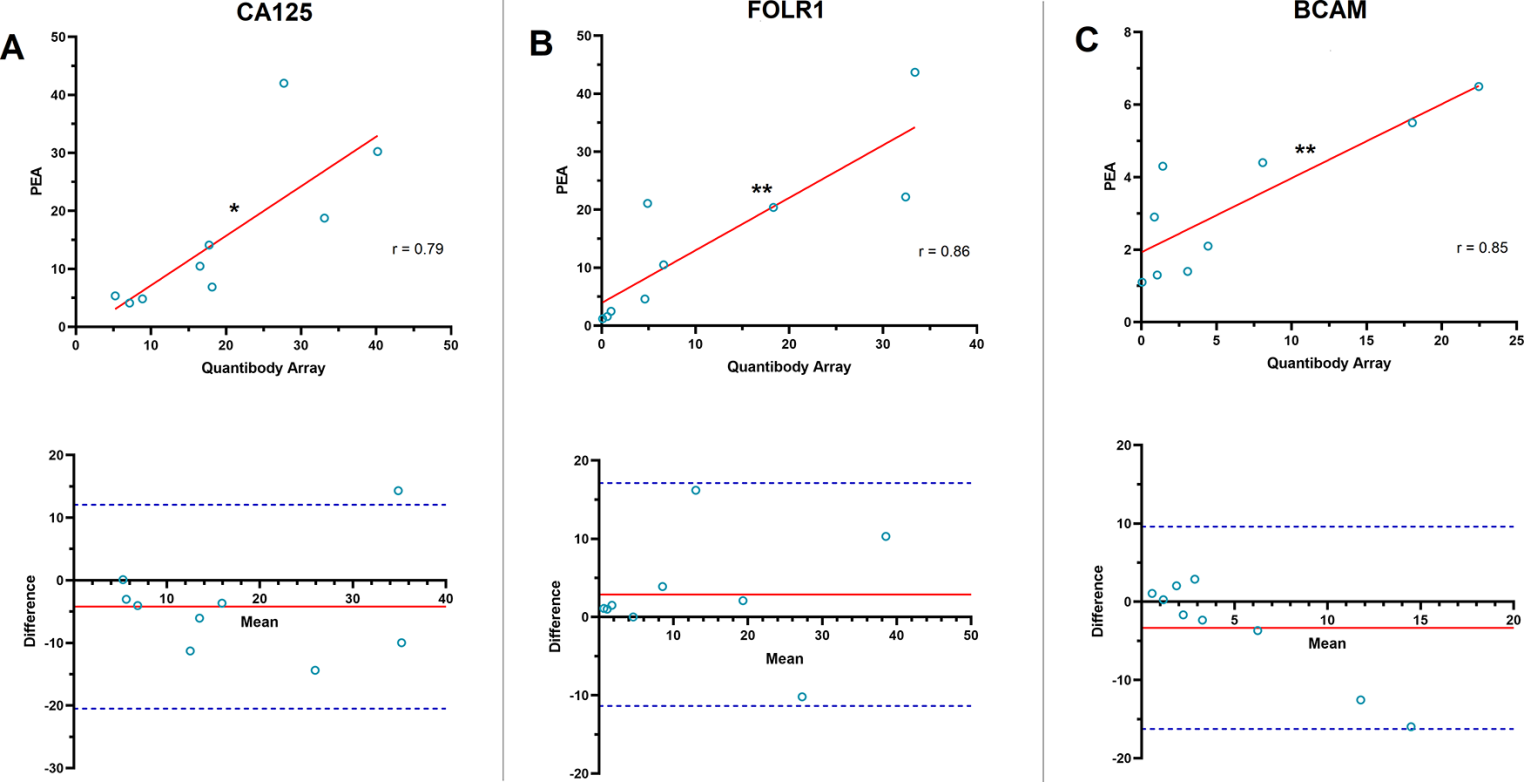
Common proteins that decreased in post-surgical serum in proximity extension assay and Quantibody array measurements. A) (Top) CA125 showed a significant correlation in the post-surgical fold change obtained from proximity extension assay (PEA) and Quantibody array in 9 ovarian cancer patients (Pearson correlation coefficient (r) = 0.785, *P = 0.0121). Solid red line in scatterplots shows the line of best fit. (Bottom) Bland-Altman plot of the post-surgical CA125 fold change measured by PEA and Quantibody array. The solid red line shows the mean difference or bias in fold change assessments (−4.21). Dashed blue lines show the upper and lower limit of agreement (LOA, Mean ± 1.96 SD; −20.50 to 12.08). B) (Top) Folate receptor 1 (FOLR1) showed a highly significant correlation in the post-surgical fold change assessed by PEA and Quantibody array (r = 0.861, **P = 0.0028). (Bottom) Bland-Altman analysis of the post-surgical FOLR1 fold change observed by PEA and Quantibody array. Solid red line shows bias (2.88). Dashed blue lines show the upper and lower LOA (−11.36 to 17.12). C) (Top) Basal cell adhesion molecule (BCAM) also showed a highly significant correlation in the post-surgical fold change assessed by PEA and Quantibody array (r = 0.845, **P = 0.0041). (Bottom) Bland-Altman plot of the post-surgical fold change in BCAM observed by PEA and Quantibody array. Solid red line shows bias (−3.33). Dashed blue lines show upper and lower LOA (−16.26 to 9.60).

Among the proteins with the largest post-surgical decrease in serum concentration, eight proteins [CA125, FOLR1, KLK11, mesothelin (MSLN), basal cell adhesion molecule (BCAM), trophoblast antigen 2 (TROP2), HE4, and cartilage oligomeric matrix protein (COMP)], were targeted by both PEA and QKA. There were significant correlation between the platforms in the post-surgical FC observed for CA125 (r = 0.785, P = 0.0121), FOLR1 (r = 0.861, P = 0.0028), and BCAM (r = 0.845, P = 0.0041) ( Figure 3). KLK11, MSLN, TROP2, HE4, and COMP did not show a significant correlation for the post-surgical FC. Bias in CA125 FC was at −4.21, where PEA data showed lower FC overall, compared to QKA ( Figure 3A). Measurement of one patient (P4) was outside of the LOA (−20.50 to 12.08). Bias in FOLR1 FC was at 2.88, with all data within the LOA range of −11.36 to 17.12 ( Figure 3B). BCAM FC showed a bias of −3.33, LOA, where all measurements were within the LOA range of −16.26 to 9.60 ( Figure 3C).

### Orthogonal validation of changes in serum protein concentration with ELISA

**Figure 4.**
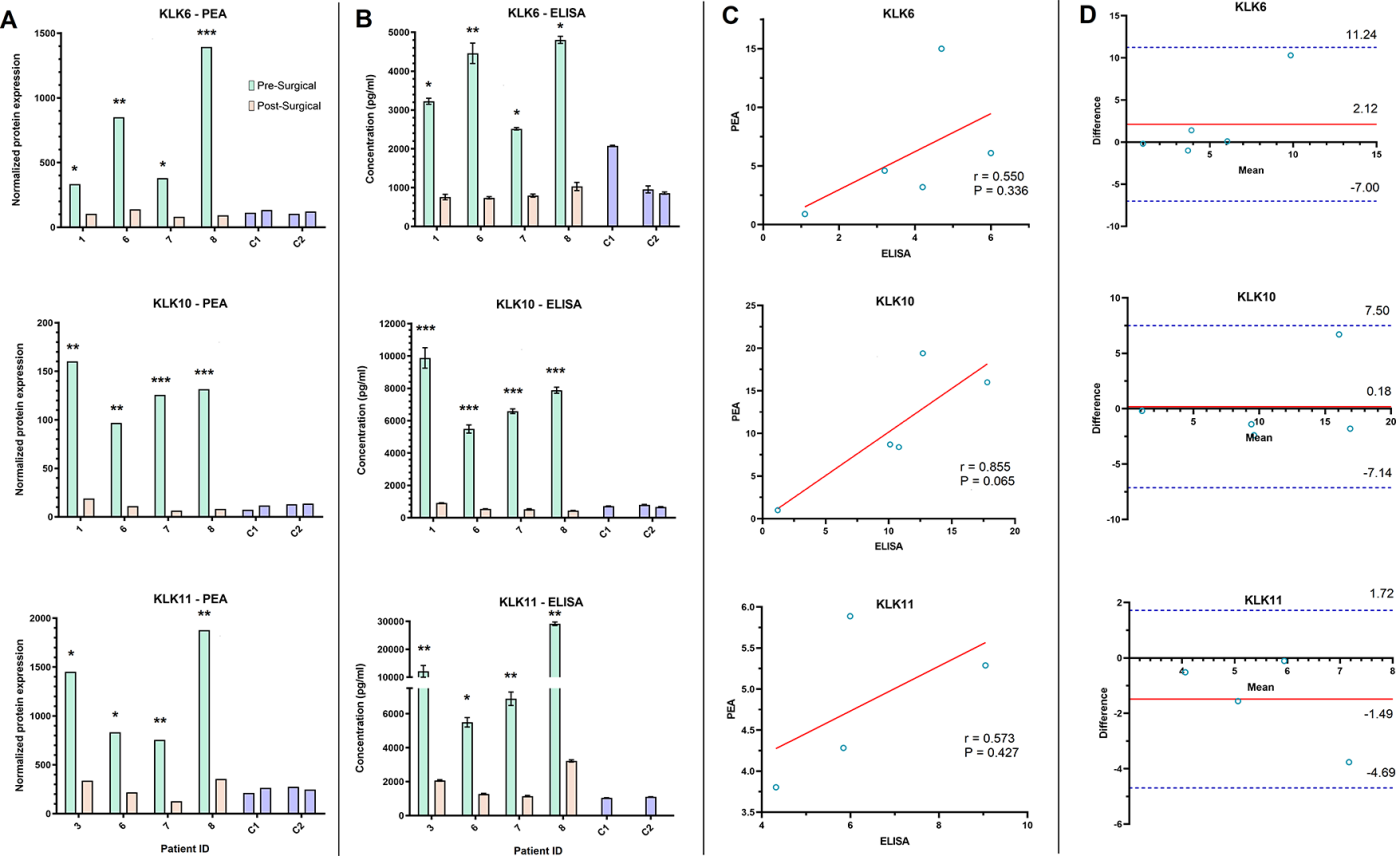
Orthogonal validation of proximity extension assay (PEA) using single target ELISA. A-B) Bar graphs depict the pre- and post-surgical serum level of three proteins (Kallikrein (KLK) 6, 10, 11) in four ovarian cancer patients and two controls (C1 and C2), as assessed by PEA technology (A) and ELISA (B). C) Scatterplot analysis of the post-surgical fold change of KLK6, 10, and 11 observed by PEA and ELISA showed a weak correlation. Solid red line in scatterplots shows the line of best fit. D) Bland-Altman plot of the post-surgical fold change in KLK6, 10, and 11 assessed by PEA and ELISA. Solid red line shows the mean difference or bias in fold change assessments. Dashed blue lines show the upper and lower limit of agreement (Mean + 1.96 SD). * Post-surgical decrease of at least two-fold. **Post-surgical decrease of at least five-fold. ***Post-surgical decrease of at least ten-fold.

Both the PEA and ELISA data showed that KLK6, 10, and 11 decreased by at least two-fold in the post-surgical samples of four patients ( Figure 4A and B). However, the correlation between the FC in KLKs shown by the two methods was not significant ( Figure 4C), with KLK10 relatively showing the strongest correlation (r = 0.855, P = 0.065). The Bland-Altman plot demonstrated minimal difference between the average of the FC obtained by PEA and QKA data for all three proteins. All data points for FC as measured by the two methods were also within the upper and lower limits of agreement (LOA), which is the 95% confidence interval that shows the maximum allowed difference between two methods ( Figure 4D).

**Figure 5.**
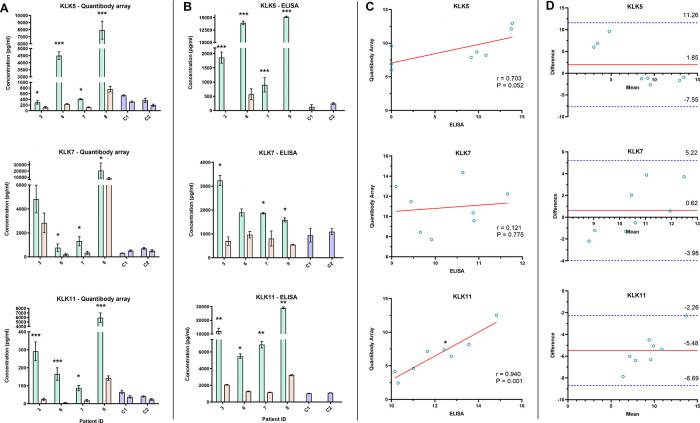
Orthogonal validation of Quantibody array using single target ELISA. A-B) Bar graphs depict the serum level of three proteins (Kallikrein (KLK) 5, 7, 11), pre- and post-surgery, in four ovarian cancer patients and two controls (C1 and C2), as assessed by Quantibody array (A) and ELISA (B). C) Scatterplot analysis of the post-surgical fold change of KLK5 and 7 observed by PEA and ELISA showed a weak correlation, while KLK11 showed a significant correlation (Pearson correlation coefficient (r) = 0.940, *P = 0.001). Solid red line in scatterplots depicts the line of best fit. D) Bland-Altman plot of the post-surgical fold change in KLK5, 7, and 11 measured by Quantibody array and ELISA. Solid red line demonstrates the mean difference or bias in fold change assessments. Dashed blue lines show the upper and lower limit of agreement (Mean ± 1.96 SD).

The QKA and ELISA results for KLK5, 7, and 11 showed at least a two-fold decrease in post- surgical samples of all four patients (except KLK7 in P3 in the QKA data and P6 in the ELISA data) ( Figure 5A and B). As both methods yield absolute protein quantitation, we correlated their serum concentration (in pg/mL) in the pre- and post-surgical samples in four patients (N = 8 samples, Figure 5C). KLK5 and KLK7 did not show a significant correlation, while KLK11 showed a significant correlation in serum concentration measured by the two methods (r = 0.940, P = 0.001). Bland-Altman analysis showed bias in FC for all three proteins ranging from -5.48 to 0.62 between the QKA and ELISA results, with all data within the LOA range ( Figure 5D).

## Discussion

The dynamic progression of cancer gives rise to a non-uniform dispersal of molecularly distinct cell subpopulations across and within tumors. ^1^ This innate cancer heterogeneity is manifested through alterations in protein expression within the tumor and it is reflected in the composition of tumor-related proteins that are released into the bloodstream. Investigation of the blood proteome of individual cancer patients is hence a non-invasive modality to uncover the tumor-derived proteins and their distinctive tumor makeup. Latest innovations in multiplex proteomics technologies have become attractive tools for mapping the circulating cancer proteome, with the hopes of giving novel insight into tumor progression and discovery of potential biomarkers to improve clinical management. ^16^ As the concept of personalized tumor markers were kept in mind, ^6, 7^ we aimed to compare the feasibility of two fundamentally different targeted multiplex proteomics technologies, PEA and QKA, to measure serological proteins that may correspond to tumor burden in specific patients. To accomplish this aim, we employed the PEA and QKA technologies to simultaneously measure 1073 and 1000 proteins, respectively, in before and after surgical sera from nine OC patients (18 samples total), with two control duplicates (4 samples in total). We compared the reliability of the two methods for detecting changes in serum protein concentration in relation to an abrupt change in tumor burden, which was achieved by tumor debulking surgery.

### Technical comparison of the two multiplex proteomics technologies

We previously published the intra-assay reproducibility of the PEA and QKA technology, finding that PEA showed high precision with a strong correlation between the technical duplicate measurements for the two controls (r = 0.95 to 0.97). ^10^ Our previous evaluation of QKA demonstrated lower precision and a weaker correlation between the technical duplicates (r = 0.853 to 0.862). ^12^ A closer look at intra-assay variation observed that 97.6% of the 1073 proteins analyzed by PEA showed a favorable coefficient of variation (CV) below 20%, 89.3% had a CV below 10%, and 0.3% showed a CV over 100% (data not shown). In contrast, 67.9% of the 1000 proteins assayed by QKA showed a CV below 20%, 47.6% had a CV below 10%, while 20.5% exhibited high variation with a CV over 100% (data not shown). We also assessed the concordance of the multiplex measurements and clinical values for CA125. The Post-surgical FC in CA125, derived from both multiplex assays, did not have a significant correlation with the clinical CA125 trends. PEA and QKA showed much lower FC in CA125 post-surgery compared to the clinical data, with the mean differences of -9.64 and -5.43–fold. Because the PEA and QKA assays measure in 92-plex and 40-plex respectively with ultrasensitivity (down to fg/mL – pg/mL), there may be a bias for low abundance proteins, while highly abundant proteins, such as large quantities of CA125 secreted by OC tumors, may be beyond the upper limit of the dynamic range. The Hook effect can occur in these situations, where very high concentrations of an analyte can cause excessive binding to both the capture and detection antibodies, thereby obstructing the antibodies from forming a sandwich immunocomplex. ^17^ This causes assay signals to decrease at a very high analyte concentrations, leading to a misconceived lower serum level. ^17^ Although we tried to correct for the Hook effect seen in three proteins, CA125, HE4 and FOLR1, in the PEA analysis after re-running sera at 1:10 dilution, some pre-surgical samples may have benefited from further dilution such as at 1:50 or 1:100, to elucidate the true post-surgical FC which would correspond better to clinical patterns. Although a previous study reported a strong correlation between CA125 concentrations as measured by PEA and clinical CA125 concentrations, ^18^ we did not see this result. This is likely because we selected for advanced OC patients whose pre-surgical clinical CA125 values exceeded 1000 U/mL, which we found showed Hook effect. Future considerations for avoiding the possible Hook effect when using multiplex proteomics technologies, such as PEA and QKA, is to analyze pre-surgical samples that may contain high concentrations of tumor markers at varying dilutions. However, this will increase the cost of analysis.

We next assessed the concordance of measurements between the two platforms and observed a significant correlation between the post-surgical FC of the 444 proteins measured by both methods. PEA and QKA showed a comparable FC in the overlapping proteins, with mean differences of −0.68–fold. We noted 36.9% (164/444) of the overlapping proteins are covered by the PEA panels that require pre-dilution of plasma/serum (from 1:10 up to 1:2025) to enrich high abundance proteins. The other overlapping proteins are in PEA panels that use undiluted plasma/serum and encompass mainly low abundance proteins. In our analysis, the PEA and QKA results showed a correlation for a range of low and high abundance proteins.

### Detecting serum proteins with the largest concentration changes that may correspond to tumor burden

Upon the selection of proteins that may correlate to tumor burden, we considered the intra-individual FC in protein concentration observed from the pre- to post-surgical samples in each patient. We propose that protein levels which decrease significantly after tumor debulking may be informative of tumor burden in the patient. The PEA data revealed 15 proteins (including CA125 and the FDA-approved marker HE4) that had at least two-fold decrease in their serum concentrations in at least six of the nine patients.CA125, FOLR1, and KRT19 had the highest median FC ( Table 1). Each patient exhibited a unique protein tumor signature consisting of 7 to 14 proteins that decreased by at least two-fold post-surgery, which may be informative of their heterogenous tumor ( Table 1). We previously employed an integrated approach that had combined proteomics, transcriptomics, and bioinformatics to identify FOLR1, as a new OC biomarker. ^19, 20^ Since then, a FOLR1-targeting antibody-drug conjugate has shown encouraging efficacy. ^21, 22^ Interestingly, tumors with overexpression in FORL1 and KRT19 have indicated increased sensitivity to cisplatin treatment, the main form of chemotherapy in OC, ^23, 24^ which could serve as predictive biomarkers of drug response. A recent study integrating PCR arrays, genome-wide microarray, and proteome analyses reaffirmed KRT19 and MSLN as OC-related proteins. ^25^ Another OC study confirmed the overexpression of KRT19 and epithelial cell adhesion molecule (EPCAM) mRNA in circulating tumor cell-fraction samples, compared to peripheral blood samples. ^26^ KRT19 is also known as CYFRA21-1 and has been extensively studied as a prognostic biomarker for OC that is complementary to CA125. ^27, 28^ Our PEA analysis yielded four KLKs (KLK6, 8, 10, 11) that had a considerable decrease in 67-78% of OC patients after surgery. Over the past decades, we have discovered these KLKs as new OC biomarkers, demonstrated their link to shorter progression-free-survival, and highlighted their potential as targets for novel therapeutics. ^29– 31^ Mainly, KLK6 is recently considered as a promising target for inhibition in OC. ^32^ Among the other proteins in Table 1, poly [ADP-ribose] polymerase 1 (PARP-1) is extensively studied for promoting angiogenesis and invasion in OC, and it is the target for novel PARP-1 inhibitors used as maintenance therapy for OC. ^33– 35^ A multi-platform analysis using PEA to measure 368 plasma proteins identified that CA125, KLK11, MSLN, KLK6, BCAM, KLK8, and HE4 among 176 proteins were elevated in OC compared to non-malignant gynecologic disorders. ^36^ The PEA results were independently validated using ELISA and an aptamer-based multiplex proteomics assay, SOMAscan, demonstrating high concordance. ^36^ Finally, glycodelin (PAEP) and TROP2 have been shown to promote proliferation and invasion of OC cells and are prognostic markers for poor OC outcome. ^37– 40^ A novel antibody-drug conjugate targeting TROP2 is currently under validation for inhibiting OC tumor growth. ^41, 42^ Although interferon lambda 1 (IFNL1) has not been studied in relation to OC thus far, this new class of interferons showed anti-tumor properties, and has been scrutinized as a new immunotherapy agent for certain types of malignancies. ^43, 44^ Overall, the PEA analysis of pre- and post-surgical samples in individual OC patients was able to identify proteins with clinical importance. As new immunotherapies and targeted therapies are revealed, ^45, 46^ our findings suggest value in further investigations into a panel of personalized tumor markers, which may include proteins with therapeutic relevance such as FOLR1, KRT19, KLK6, PARP-1, TROP2, and IFNL1. This could be an instrumental tool for identifying the heterogenous makeup of individual tumors to select the best treatment options for individual patients.

The QKA data indicated 11 proteins (including CA125) with at least two-fold serum concentration decrease, in at least six of the nine patients ( Table 2). Each patient showed a distinct protein tumor signature consisting of 4 to 10 proteins that showed at least two-fold post-surgical decrease, which may be informative of their tumor burden ( Table 2). From the candidate proteins that were selected by separate PEA and QKA analysis, there were eight candidate proteins in common. However, only CA125, FOLR1 and BCAM from the eight common candidate proteins showed significant correlation in the trend of post-surgical FC ( Figure 3), although the lack of statistical significance in other proteins could be due to the limited sample size, since KLK11 was selected as a top protein by both analyses ( Tables 1 and 2). In fact, the three proteins that showed the highest median FC based on QKA analysis, (CA125, KLK11, and FOLR1) were also selected in the PEA analysis. QKA data also identified two other KLKs (KLK5 and 7), which we previously discovered to be indicators of poor prognosis in OC. ^47– 49^ Recent clinical and tumor-biological studies also recognized KLK5 and 7 as favorable drug targets for OC. ^32^ Among the other proteins in Table 2, renin has been studied in relation to an increased activity in the hormonal renin-angiotensin system in OC. ^50^ However, extrarenal renin-secreting tumors represent a subpopulation of OC tumors. ^51^ DNA microarray analysis has revealed upregulation of activin receptor type 1 (ACVR1) in OC tissue compared to normal ovarian tissue. ^52^ Furthermore, the binding of stress-induced phosphoprotein 1 (STIP1) to ACVR1 and bone morphogenetic protein 9 (BMP9) signaling of ACVR1 have both shown to promote OC proliferation. ^53, 54^ Notably, microarray analysis of OC tumor tissue revealed just 29% of tumors expressed BMP9, which induces ACVR1 pathway hyperactivation. ^54^ In recent years, unravelling the over-expression of nicotinamide phosphoribosyltransferase (NAMPT), a critical enzyme in the NAD salvage pathway, had prompted the development of new therapeutic strategies for NAMPT inhibition in subsets of OC patients. ^55, 56^ The immunosuppressive receptor, signaling lymphocytic activation molecule (SLAM), has also shown heterogeneous expression, where it was overexpressed in a subset of CD3+ T lymphocytes isolated from the blood of OC patients. ^57^ One study thus far has described an upregulation of COMP in OC through cancer stem cells analysis. ^58^ Nevertheless, COMP has been widely studied for inducing cancer stem cell formation and proliferation, ^59, 60^ as well its correlation with poor survival and recurrence in breast cancer, colon cancer, and prostate cancer. ^59, 61, 62^ Finally, one study linked toll-like receptor 1 (TLR1), a pattern recognition receptor for innate immunity, to inflammation in the OC microenvironment. ^63^ Remarkably, several studies have uncovered that genetic polymorphisms in the toll-like receptor gene cluster (TLR6, TLR1, and TLR10) promote chronic inflammation and enhance prostate cancer risk, in a subpopulation of individuals, ^64, 65^ warranting further investigation in other cancer subpopulations. Collectively, the QKA analysis was able to identify proteins associated with OC and tumorigenesis, where many proteins exhibited varied upregulations in subsets of OC patients. Our results support the idea that a panel of tumor-related proteins, including heterogeneously expressed proteins such as renin, ACVR1, TLR1, and KLKs, ^5, 7, 11^ may encompass tumor heterogeneity in ovarian cancer patients.

In order to assess the concordance of the multiplexed data with our validated, in-house ELISAs, we performed single-target ELISA assays to quantitatively measure the change in serum expression levels of five proteins (KLK5, 6, 7, 10, and 11), in the same four patients and two controls. The two multiplex assays showed at least two-fold post-surgical decrease in serum concentrations of all KLKs ( Figures 4 and 5). However, only KLK11 measurements by QKA showed statistical significance for correlation with ELISA ( Figures 4 and 5), which could be due to limitations in our sample size. Visually assessing the agreement in multiplexed measurements with ELISA assays revealed that PEA, compared to QKA, showed a lower mean difference in post-surgical KLKs FC in the ELISA results (bias ranged from -1.49 to 0.18-fold for PEA versus -5.48 to 0.62-fold for QKA) ( Figures 4 and 5). Two studies have reported a favorable correlation of PEA data and ELISA for validating candidate cancer-related proteins in OC and glioma. ^36, 66^ One recent study had employed Quantibody arrays, akin to QKA, for the identification of differentially expressed plasma proteins in OC, which resulted in validating nine candidates successfully by using ELISA. ^67^ However, we previously reported discrepancies between QKA and ELISA results for validating nine candidate proteins that may correlate to tumor burden in pancreatic cancer patients. ^12^ The analytical precision of multiplexed platforms may be highly analyte-dependent, and multiplexed data may benefit from independent validation to verify the protein quantitation.

## Conclusions

Our preliminary investigation demonstrated the initial potential of two targeted multiplex proteomics platforms, PEA and QKA, for identifying changes in serum protein concentrations that may associate with tumor burden in OC patients. While multiplex technologies possess high sensitivity, which is key to discovering clinically relevant proteins that are present at extremely low concentrations in the plasma/serum, there may be a concern for missing highly abundant tumor-derived proteins, such as CA125 for OC, which increase by several orders of magnitude in cancer plasma/serum. This bias can generally be avoided by analyzing serum at varying dilutions to enhance accurate detection of high abundance analytes. Although our study represented a limited sample size and cannot conclusively determine the technical reliability of the PEA and QKA assays, the multiplexed data in general, were in concordance with each other and, single target ELISAs for identifying general patterns of large changes in serum protein concentration. Our results showed the multiplatform approach was complementary for detecting proteins that were associated with pathobiological and clinical significance for OC, including encompassing a heterogenous array of known OC biomarkers, new therapeutic targets, and emerging markers of polymorphic gene/protein expression. Each patient may bear a unique tumor protein signature, a concept that warrants future investigation in larger cohorts. In the future, personalized tumor protein profiles could be monitored for changes in expression that may trigger clinically actionable events, such as selecting the best treatment, calculating prognostic risk, and detecting cancer recurrence in the patient. Our conclusions from a small sample size suggest that novel targeted proteomics methods can serve as valuable complementary tools to each other for comprehensively identifying diverse tumor protein signatures in the cancer proteome. However, it is beneficial to corroborate the data with independent, orthogonal methods such as MS and ELISA for accurate and validated protein quantitation, in order to minimize false results and promote further advancements in the biomarker discovery pipeline.

## Data availability

### Underlying data

Harvard Dataverse: Comparison of two multiplexed technologies for profiling >1,000 serum proteins that may associate with tumor burden – a pilot study

https://doi.org/10.7910/DVN/KRROIC. ^11^

This project contains the following underlying data:

1. Clinical CA125 vs Olink and Raybiotech (CA125 measurements from clinical assay, PEA technology and QKA)
2. ELISA raw data results for KLK5, 7, 11 (KLK5, 7, 11 ELISA raw data)
3. ELISA raw data results for KLK6 and KLK10 (KLK6 and KLK10 ELISA raw data)
4. Raw data from Olink_1 in 10 dilution of Oncology panel (PEA technology data for 92 proteins with sample 1:10 dilution)
5. Raw data from Olink_1076 unique protein measurements (PEA technology data for 1076 unique proteins)
6. Raw data from Raybiotech_1000 unique protein measurements (QKA data for 1000 proteins)

Data are available under the terms of the CC BY-NC-SA (CC0 1.0 Universal).

### Extended data

Harvard Dataverse: Extended data for Comparison of two multiplexed technologies for profiling >1,000 serum proteins that may associate with tumor burden

https://doi.org/10.7910/DVN/7C3SV0. ^15^

This project contains the following extended data:

• Extended table 1. (List of overlapping protein targets for proximity extension assay and Quantibody ^®^ Kiloplex Array).
