# Supplementary material for: Comparison of two multiplexed technologies for profiling >1,000 serum proteins that may associate with tumor burden
Source: F1000Res. 2021 Jun 28;10:509. [Version 1] doi: 10.12688/f1000research.53364.1 (PMC8609392; doi:10.12688/f1000research.53364.1)
Supplement: Peer review history

# Peer review: Reviewer response for version 1

**DOI:** 10.5256/f1000research.56737.r88484

**Published:** 2021-11-22

**Authors:** Stefan Holdenrieder

**Affiliations:** 1 Institute for Laboratory Medicine, German Heart Centre, Technical University Munich, Munich, Germany

In the present pilot study, the authors compared two multiplexed technologies (proximity extension assay, PEA and quantibody kiloplex array, QKA), for profiling a large number of serum proteins. Tested samples were collected prior and after surgery of ovarian cancer patients. Thus, the authors hypothesized that markers with significant decreases in serum concentrations were associated with tumor burden.

 However, they clearly state that they performed this study only as preliminary investigation justifying the low number of patients and controls. While this limits the findings substantially, great changes in marker concentrations may indicate candidate markers that should be further validated.

 Within the large number of markers (>1000) measured by both proteomics technologies, more than 400 were overlapping. The most relevant one, the ovarian cancer marker CA 125, showed only weak correlation between both methods, and also the postsurgical changes in the 400 common markers were only weakly correlated. Among all markers, 15 showed an at least two-fold postsurgical decrease in two thirds of the patients by the use of PEA and 11 by the use of QKA; 8 of them were detected by both methods of which only 3 showed correlations in postsurgical changes by PEA and QKA. Subsequently, 5 kallikrein markers were validated by ELISA. The postsurgical changes correlated with PEA for 3 markers, absolute values only for one.

 The authors conclude that both novel targeted proteomics platforms are promising tools for identifying candidate serological tumor-related proteins. However, they also admit several limitations of their study and some further ones have to be added:

- The small number of patients and controls that could lead to missing or overestimating single markers.
- The lack of sufficient reproducibility at least for one platform.
- The only weak correlation between both platforms.
- The need for different dilutions and the challenge to identify and avoid hook effects.
- The difference in absolute value levels for markers if different platforms were used.
- The weak correlation results in the validation experiment with single markers ELISAs.

Obviously, the authors are aware of the limitations and the long way to identify and validate new diagnostic markers for ovarian cancer. They are encouraged to do these validation steps on larger cohorts with proper assays for the new markers identified by their proteomics approach in a subsequent study.

Is the work clearly and accurately presented and does it cite the current literature?

Yes

If applicable, is the statistical analysis and its interpretation appropriate?

Yes

Are all the source data underlying the results available to ensure full reproducibility?

Yes

Is the study design appropriate and is the work technically sound?

Yes

Are the conclusions drawn adequately supported by the results?

Yes

Are sufficient details of methods and analysis provided to allow replication by others?

Yes

Reviewer Expertise:

Biomarkers in cancer diagnostics

I confirm that I have read this submission and believe that I have an appropriate level of expertise to confirm that it is of an acceptable scientific standard.

# Peer review: Reviewer response for version 1

**DOI:** 10.5256/f1000research.56737.r88486

**Published:** 2021-11-11

**Authors:** Anna E Lokshin

**Affiliations:** 1 Department of Medicine, University of Pittsburgh Medical Center, Pittsburgh, PA, USA

- This study is the first**** to compare two targeted multiplex proteomics technologies, proximity extension assay (PEA) and Quantibody® Kiloplex Array (QKA), for analysis of serum protein concentration changes.
- The results show that the data obtained by platforms highly correlate. Authors have identified proteins that decrease following debulking surgery for ovarian cancer.
- Data were validated by ELISA. This study is of great importance for future biomarker research using highly novel targeted proteomics platforms. 

Is the work clearly and accurately presented and does it cite the current literature?

Yes

If applicable, is the statistical analysis and its interpretation appropriate?

Yes

Are all the source data underlying the results available to ensure full reproducibility?

Yes

Is the study design appropriate and is the work technically sound?

Yes

Are the conclusions drawn adequately supported by the results?

Yes

Are sufficient details of methods and analysis provided to allow replication by others?

Yes

Reviewer Expertise:

Cancer biomarkers

I confirm that I have read this submission and believe that I have an appropriate level of expertise to confirm that it is of an acceptable scientific standard.

# Peer review: Reviewer response for version 1

**DOI:** 10.5256/f1000research.56737.r89025

**Published:** 2021-07-28

**Authors:** Nivedita Chowdhury

**Affiliations:** 1 Atreca Inc, San Carlos, CA, USA

Ren *et al.* have demonstrated a comparative study of identifying serum proteins that may associate with tumor burden in ovarian cancers, using two multiplexed proteomic technologies. This is an interesting study and the authors have elucidated the two methods distinctly in a patient cohort. Overall, the paper is well written, researched and structured. However, there are a few shortcomings, especially the justification for choosing the two methods and the clinical relevance. I also find that the authors have not stressed on the novelty of this study enough. Below, I have provided numerous comments on the text and suggestions for more in-depth analysis of the data. Given these, the manuscript requires major revisions.

**Abstract:**

- Well summarized in terms of the methodology including validation using ELISAs. However, please use assertive language. Use of "pilot" and "preliminary" is not only redundant but also indicates low conviction/doubts on their own study.  Also, change “expect that proteins have decreased…”. For eg. “speculate/hypothesize that proteins with reduced concentrations in the post-surgery sera, correlate to tumor burden in each patient”.
- In the Results section, the authors need to emphasize on the novel findings of serum proteins previously not associated with tumor burden and/or OC. Also remove “novel proteomic platforms” from the conclusion - PEA is fairly well known and a few groups, including the authors' have published QKA results in the past.

**Introduction:**

 The authors have included most of the relevant background with respect to tumor-associated proteins, the two technologies, drawbacks of the conventional ELISA/MS methods and OC. They also link their previous work to show a natural progression to their current work. The research aim has been clearly outlined. I have two comments:

1. Add more about other methods currently used to profile serum concentrations, including multiplex technologies. Eg. SOMAscan. Include their limitations and justify your choice of testing PEA and QKA only - if they are more sensitive, specific, personalized, comprehensive, cost-effective, etc. If they are highly personalized, how do they compare to proteomics including single-cell methods such as CyTOF?
2. Please add more relevant references for other techniques such as Gool *et al.* (2020 ^1^) and Makridakis *et al*. (2020 ^2^). Please also include more information on the already established circulating tumor markers in OC such as EPCAM, MUC1, etc. Also, include a reference for Paragraph 1, Line 7, "Tumors secrete a medley…".

**Methods:**

- The study is well designed to address the research objectives. The methods are described in detail and the process of subject selection is well-outlined. I am interested in knowing about the criteria for selection fo the 5 proteins to validate using ELISA. Since there was an overlap of many proteins between the two technologies, on what basis were the 5 chosen for further validation?

**Results and discussion:**

 The results are stated clearly and the figure legends are detailed and self explanatory. I have the following questions:

1. Considering that the pearson coefficient, r > 0.7 is considered strong positive correlation, how do your results add to what was previously known? Other groups (that you have kindly referenced) have shown high correlation in their findings with clinical values. Moreover, your previous work (Ren *et al.*, 2020 ^3^) also showed no-to-low correlation in a different tumor - please elaborate on the possible reasons behind this discrepancy, besides small patient cohort.
2. The conclusions the authors draw about the decrease in proteins after surgery being related to tumor burden may be overestimated. In the methods, it is stated that the patients were also given adjuvant chemotherapy. We know that chemotherapy can result in changes in extra-cellular matrix proteins and in those related to inflammation. How are the authors certain that the changes observed are as a result of surgery alone, and thus, relate to tumor burden?
3. Is there a way to demonstrate clinical relevance of this study? Since this is a retrospective study of patients with advanced disease, the survival data may be available. I suggest the authors do a survival analysis of selected proteins to check for their correlation with survival or progression. These results can then be further validated in a larger, publicly available dataset of serum proteins. If a correlation between them and clinical outcome is observed, it lends more credibility to the authors' claim.

 

 Lastly, I have the following comments on the paper as a whole:

1. The limitations addressed in the conclusion are great opportunities to inform further research. They also shed light on possible bias based on abundance of proteins. The authors' efforts to control for this are commendable!
2. Please stress on the novelty of this study more! For eg. in the discussion, the authors elude to the various functions of the proteins previously identified in OC and other tumors. Explain how this is a novel finding using these platforms and how it can help change the field to move towards similar technologies. Also, the authors identify FOLR1, which could be a novel finding in relation to OC prognosis.
3. The title can be better phrased. For eg. "Evaluation and comparison of two multiplexed platforms profiling serum proteins and its possible correlation with tumor burden (or clinical outcome) in human ovarian cancer."

Is the work clearly and accurately presented and does it cite the current literature?

Partly

If applicable, is the statistical analysis and its interpretation appropriate?

Yes

Are all the source data underlying the results available to ensure full reproducibility?

Yes

Is the study design appropriate and is the work technically sound?

Yes

Are the conclusions drawn adequately supported by the results?

Partly

Are sufficient details of methods and analysis provided to allow replication by others?

Yes

Reviewer Expertise:

Single-cell technologies, tumor biology, biomarkers, tumor immunology, histology, translational sciences.

I confirm that I have read this submission and believe that I have an appropriate level of expertise to confirm that it is of an acceptable scientific standard, however I have significant reservations, as outlined above.

## References

[1] : Analytical techniques for multiplex analysis of protein biomarkers. *Expert Rev Proteomics* .17(4) : 10.1080/14789450.2020.1763174 257-273 10.1080/14789450.2020.1763174 32427033

[2] : Multiplexed MRM-based protein quantification of putative prognostic biomarkers for chronic kidney disease progression in plasma. *Scientific Reports* .2020;10(1) : 10.1038/s41598-020-61496-z 10.1038/s41598-020-61496-z 32179759PMC7076027

[3] : Investigating a novel multiplex proteomics technology for detection of changes in serum protein concentrations that may correlate to tumor burden. *F1000Res* .2020;9: 10.12688/f1000research.24654.2 732 10.12688/f1000research.24654.2 33274048PMC7682495
